# Clinical and Invasive Hemodynamic Correlates of the TAPSE/sPAP Ratio in Pulmonary Hypertension

**DOI:** 10.1002/clc.70520

**Published:** 2026-11-09

**Authors:** Salim Yaşar, Özkan Eravci, Cemal Dinç, Ahmet Faruk Yağci, Selen Eşki, Erkan Yildirim, Suat Görmel, Murat Çelik

**Affiliations:** ^1^ Department of Cardiology, Gulhane Training and Research Hospital University of Health Sciences Ankara Türkiye; ^2^ Cardiology Clinic Dr. Yaşar Eryılmaz Doğubayazıt State Hospital Ağrı Türkiye

**Keywords:** ehocardiography, pulmonary hypertension, right heart catheterization, right ventricular–pulmonary arterial coupling, TAPSE/sPAP ratio

## Abstract

**Background:**

Right ventricular (RV) adaptation to increased pulmonary afterload is a major determinant of prognosis in pulmonary hypertension (PH), yet comprehensive validation of simple noninvasive markers against invasive hemodynamic parameters remains limited. We investigated whether the tricuspid annular plane systolic excursion to systolic pulmonary artery pressure (TAPSE/sPAP) ratio reflects the clinical, echocardiographic, and invasive hemodynamic spectrum of PH.

**Methods:**

We retrospectively analyzed 209 consecutive patients with right heart catheterization‐confirmed PH who underwent comprehensive clinical assessment, transthoracic echocardiography, and invasive hemodynamic evaluation between 2016 and 2024. Patients were stratified into quartiles according to the TAPSE/sPAP ratio, and clinical, laboratory, echocardiographic, and hemodynamic characteristics were compared.

**Results:**

The mean age was 58.8 ± 16.5 years, and 61% of patients were female. Lower TAPSE/sPAP ratios were associated with older age, higher NT‐proBNP and serum uric acid levels, and lower estimated glomerular filtration rate (all *p* < 0.05). Decreasing TAPSE/sPAP ratios were also associated with larger right atrial and right ventricular dimensions, lower TAPSE, and higher tricuspid regurgitation velocity and estimated sPAP (all *p* < 0.001). Invasive hemodynamic assessment demonstrated progressively higher right ventricular systolic pressure, mean pulmonary artery pressure, and pulmonary vascular resistance (all *p* < 0.001), whereas mixed venous oxygen saturation increased across quartiles (*p* = 0.036).

**Conclusions:**

The TAPSE/sPAP ratio is a practical, noninvasive marker that closely reflects right ventricular remodeling and invasive hemodynamic impairment in pulmonary hypertension. Lower TAPSE/sPAP ratios identify patients with more advanced RV–pulmonary arterial uncoupling and may improve bedside hemodynamic risk stratification.

## Graphical abstract

Lower TAPSE/sPAP ratios are associated with adverse clinical characteristics, progressive right ventricular remodeling, and greater invasive hemodynamic impairment in pulmonary hypertension. TAPSE/sPAP provides a simple, noninvasive marker for assessing RV–pulmonary arterial uncoupling and bedside hemodynamic risk stratification.

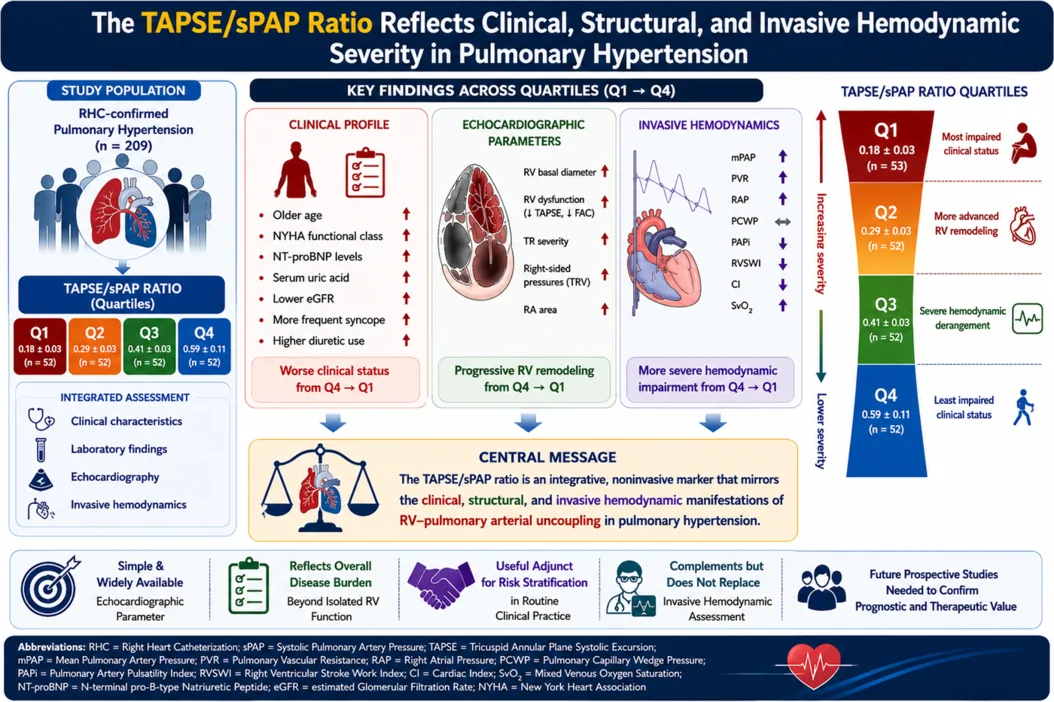

## 1 Introduction

Pulmonary hypertension (PH) encompasses a heterogeneous group of disorders in which abnormal pulmonary vascular loading imposes a sustained burden on the right ventricle (RV). As the disease progresses, clinical status is increasingly influenced by whether the RV can preserve forward flow despite the rise in afterload. Accordingly, assessment of RV adaptation, rather than pulmonary pressure in isolation, has become an important component of contemporary evaluation and risk stratification in PH [1, 2, 3].

RV‐pulmonary arterial (RV‐PA) coupling describes the relationship between RV contractile performance and the load imposed by the pulmonary circulation. Although pressure‐volume loop‐derived indices provide the most direct assessment of this interaction, their invasive and technically demanding nature limits routine use [4]. Echocardiography therefore offers clinically useful surrogate measures. The TAPSE/sPAP ratio combines longitudinal RV systolic excursion with an estimate of pulmonary arterial systolic load and has been increasingly used as a practical noninvasive index of RV‐PA interaction [4, 5].

Existing studies have largely examined TAPSE/sPAP in relation to prognosis, functional capacity, or clinical outcomes, whereas its relationship with a broad panel of simultaneously assessed invasive hemodynamic variables has been less extensively characterized [6]. Recent evidence also indicates that the clinical meaning and proposed thresholds of TAPSE/sPAP may vary across disease settings, underscoring the importance of interpreting the ratio within the relevant hemodynamic context [7].

Therefore, the present study aimed to investigate the association between the TAPSE/sPAP ratio and clinical characteristics, laboratory findings, echocardiographic parameters, and invasive hemodynamic measurements in patients with right heart catheterization‐confirmed pulmonary hypertension. We hypothesized that lower TAPSE/sPAP ratios would identify patients with more advanced right ventricular remodeling, greater pulmonary vascular burden, and more severe RV–pulmonary arterial uncoupling, supporting the use of TAPSE/sPAP as an integrative noninvasive marker for bedside hemodynamic risk assessment.

## 2 Methods

### 2.1 Study Population

This retrospective observational study included consecutive adult patients diagnosed with pulmonary hypertension (PH) who underwent comprehensive transthoracic echocardiography and right heart catheterization at our institution between January 2016 and December 2024. Pulmonary hypertension was defined according to the 2022 ESC/ERS Guidelines as a mean pulmonary artery pressure (mPAP) ≥ 20 mmHg measured during right heart catheterization. Patients with significant left‐sided valvular heart disease, advanced left ventricular systolic dysfunction, congenital heart disease requiring surgical correction, inadequate echocardiographic image quality, or incomplete clinical or hemodynamic data were excluded.

Clinical, laboratory, echocardiographic, and hemodynamic data were retrospectively retrieved from the hospital electronic medical record and automation system. Echocardiographic examinations had been performed during routine clinical care by multiple cardiologists. Because the TAPSE/sPAP quartile groups and study outcomes were defined retrospectively after data collection, the cardiologists performing the original echocardiographic assessments were unaware of the subsequent study‐group allocation and study outcomes.

Patients were stratified into quartiles according to the TAPSE/sPAP ratio. Quartile boundaries were determined from the study population, resulting in four groups with approximately equal numbers of patients. The corresponding mean ± standard deviation values for each quartile were 0.18 ± 0.03, 0.29 ± 0.03, 0.41 ± 0.03, and 0.59 ± 0.11, respectively.

The study protocol was approved by the Institutional Ethics Committee (Approval No. 2026‐131) and was conducted in accordance with the principles of the Declaration of Helsinki.

### 2.2 Echocardiographic Assessment

Comprehensive transthoracic echocardiography was performed using commercially available ultrasound systems in accordance with guideline‐based recommendations for cardiac chamber quantification and right‐heart assessment [3, 8]. Standard left ventricular and right ventricular measurements were obtained from conventional views. Right‐heart measurements were interpreted according to the American Society of Echocardiography recommendations, including the 2025 guideline addressing echocardiographic assessment of the right heart and pulmonary hypertension [8].

Left ventricular ejection fraction (LVEF) was calculated using the modified Simpson biplane method. Right ventricular systolic function was assessed by tricuspid annular plane systolic excursion (TAPSE), measured using M‐mode at the lateral tricuspid annulus. Tricuspid regurgitation velocity (TRV) was measured by continuous‐wave Doppler, and systolic pulmonary artery pressure (sPAP) was estimated using the modified Bernoulli equation after adding estimated right atrial pressure based on inferior vena cava size and collapsibility. Additional echocardiographic parameters included left atrial diameter, right atrial diameter, right ventricular basal diameter, and inferior vena cava diameter.

### 2.3 Right Heart Catheterization

Right heart catheterization was performed via the femoral venous approach using standard techniques. Hemodynamic measurements included right atrial pressure (RAP), right ventricular systolic pressure (RVSP), systolic, diastolic, and mean pulmonary artery pressures, pulmonary capillary wedge pressure (PCWP), cardiac output (CO), cardiac index (CI), pulmonary vascular resistance (PVR), systemic vascular resistance (SVR), mean arterial pressure (MAP), mixed venous oxygen saturation (MvO_2_), and femoral arterial oxygen saturation (FaO_2_). Cardiac output was determined using the thermodilution method, and pulmonary vascular resistance was calculated according to standard hemodynamic equations and expressed in Wood units.

### 2.4 Statistical Analysis

Continuous variables are presented as mean ± standard deviation, whereas categorical variables are expressed as frequencies and percentages. Comparisons among TAPSE/sPAP quartiles were performed using one‐way analysis of variance (ANOVA) for continuous variables and the chi‐square test for categorical variables, as appropriate. Statistical significance was defined as a two‐sided *p* value < 0.05. All statistical analyses were performed using IBM SPSS Statistics for Windows, Version 26.0 (IBM Corp., Armonk, NY, USA).

## 3 Results

### 3.1 Demographic and Clinical Characteristics

A total of 209 patients with right heart catheterization‐confirmed pulmonary hypertension were included in the study. The mean age was 58.8 ± 16.5 years, and 61% of the study population were female. Patients with progressively higher TAPSE/sPAP ratios demonstrated a more favorable clinical profile. Increasing TAPSE/sPAP quartiles were associated with younger age (*p* = 0.001), better renal function as reflected by higher estimated glomerular filtration rate (*p* = 0.004), lower serum uric acid concentrations (*p* = 0.010), and lower NT‐proBNP levels (*p* = 0.015). Other baseline demographic and laboratory characteristics are summarized in Table 1.

**TABLE 1:** Demographic and laboratory parameters.

Parameter	Group 1 (*n* = 53)	Group 2 (*n* = 52)	Group 3 (*n* = 52)	Group 4 (*n* = 52)	*p* value
TAPSE/sPAP ratio	0.18 ± 0.03	0.29 ± 0.03	0.41 ± 0.03	0.59 ± 0.11	< 0.001
Age (years)	63.39 ± 15.69	59.69 ± 17.63	61.13 ± 13.22	51.09 ± 15.80	0.001
BMI (kg/m^2^)	27.12 ± 7.16	30.06 ± 7.24	30.19 ± 5.12	29.07 ± 5.28	0.243
6MWD (m)	352.93 ± 108.19	363.23 ± 115.69	422.61 ± 148.95	391.94 ± 80.16	0.185
NT‐proBNP (pg/mL)	1472.73 ± 2086.19	842.76 ± 954.19	761.30 ± 1305.48	631.09 ± 1171.81	0.015
eGFR (mL/min/1.73 m^2^)	70.92 ± 24.00	84.08 ± 24.38	74.23 ± 27.54	86.26 ± 23.36	0.004
Serum uric acid (mg/dL)	6.81 ± 2.54	5.99 ± 1.94	5.87 ± 2.40	5.33 ± 1.70	0.010
Hemoglobin (g/dL)	13.11 ± 2.49	12.97 ± 2.42	12.41 ± 2.07	13.67 ± 2.19	0.051
Hematocrit (%)	40.67 ± 7.11	39.53 ± 6.83	38.14 ± 5.61	40.96 ± 6.32	0.113
Platelet (×10^9^/L)	265.52 ± 97.50	239.88 ± 65.49	234.07 ± 95.83	242.45 ± 85.32	0.267
WBC (×10^9^/L)	9.10 ± 2.91	8.16 ± 3.53	7.24 ± 2.21	7.27 ± 2.74	0.003

Abbreviations: 6MWD, 6‐min walk distance; BMI, body mass index; eGFR, estimated glomerular filtration rate; NT‐proBNP, N‐terminal pro‐B‐type natriuretic peptide; sPAP, systolic pulmonary artery pressure; TAPSE, tricuspid annular plane systolic excursion; WBC, white blood cell count.

### 3.2 Echocardiographic Findings

Progressively higher TAPSE/sPAP ratios were accompanied by consistent improvements in right ventricular structure and function. Patients in higher quartiles demonstrated significantly smaller right atrial and right ventricular dimensions together with higher TAPSE values, indicating better preserved RV systolic performance (all *p* < 0.001). Similarly, tricuspid regurgitation velocity, estimated systolic pulmonary artery pressure, and inferior vena cava diameter decreased progressively across increasing TAPSE/sPAP quartiles (all *p* ≤ 0.006). Left ventricular ejection fraction also showed a modest but statistically significant increase across groups (*p* = 0.010). Detailed echocardiographic findings are presented in Table 2, and the overall pattern of right ventricular remodeling across quartiles is illustrated in Figure 1.

**TABLE 2:** Echocardiographic parameters.

Parameter	Group 1	Group 2	Group 3	Group 4	*p* value
LVEF (%)	56.03 ± 8.93	58.88 ± 4.88	59.48 ± 5.04	59.56 ± 4.45	0.010
LA (mm)	41.13 ± 8.22	39.86 ± 7.51	42.00 ± 7.55	36.57 ± 5.59	0.001
RA (mm)	51.94 ± 10.78	45.76 ± 9.12	42.74 ± 6.87	39.80 ± 8.64	< 0.001
RVD (mm)	47.68 ± 8.60	44.35 ± 8.60	41.04 ± 6.52	37.95 ± 6.52	< 0.001
TAPSE (mm)	16.09 ± 3.50	19.90 ± 3.58	21.19 ± 3.76	22.57 ± 2.57	< 0.001
TRV (m/sec)	4.37 ± 0.49	3.66 ± 0.47	3.23 ± 0.35	2.76 ± 0.53	< 0.001
sPAP (mmHg)	90.43 ± 18.22	67.11 ± 12.58	51.78 ± 9.19	39.17 ± 8.57	< 0.001
IVC (mm)	21.25 ± 4.71	19.30 ± 4.70	16.13 ± 7.68	15.93 ± 5.33	0.006

Abbreviations: IVC, inferior vena cava; LA, left atrium; LVEF, left ventricular ejection fraction; RA, right atrium; RVD, right ventricular diameter; TAPSE, tricuspid annular plane systolic excursion; sPAP, systolic pulmonary artery pressure; TRV, tricuspid regurgitation velocity.

**FIGURE 1:**
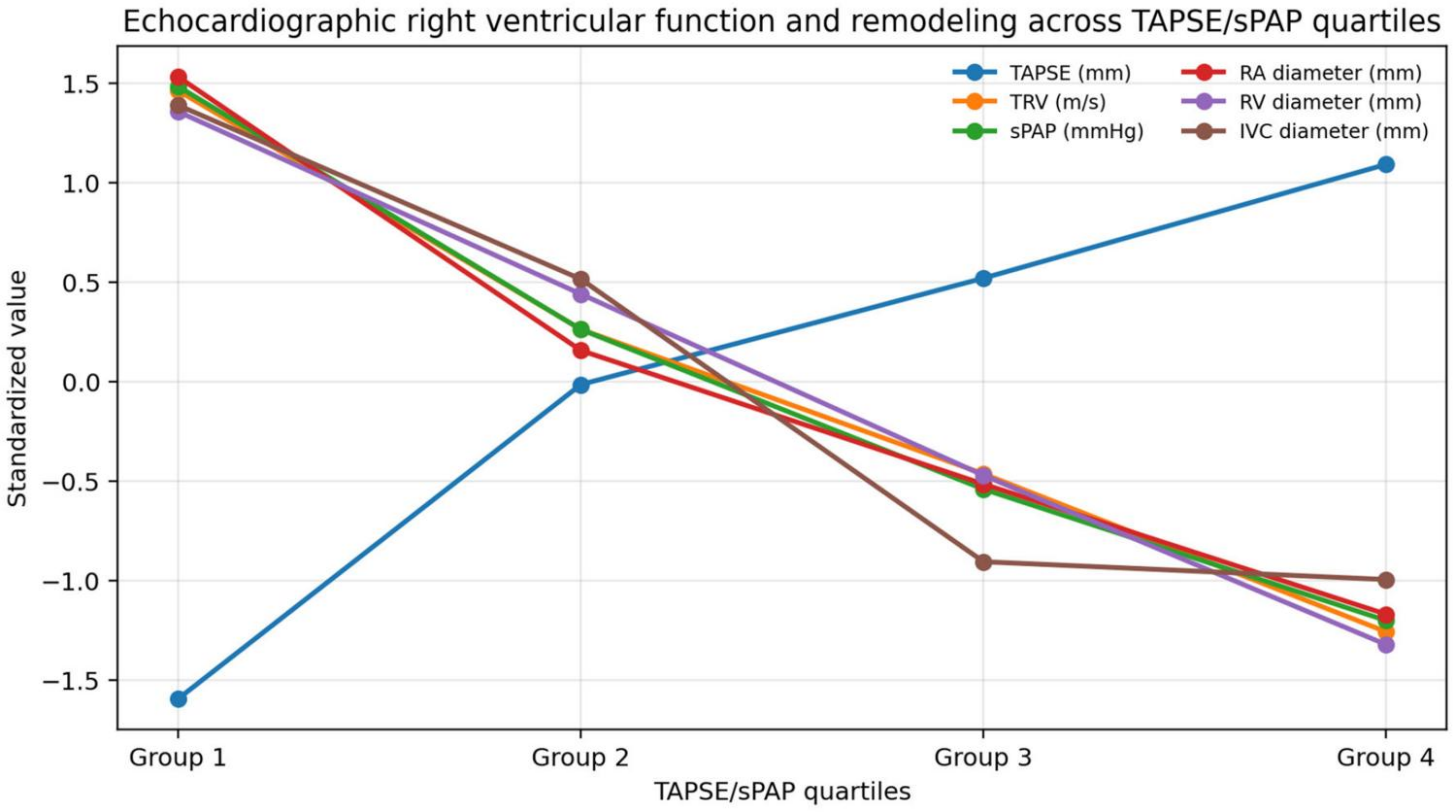
Echocardiographic right ventricular function and remodeling across TAPSE/sPAP quartiles. Tricuspid annular plane systolic excursion (TAPSE), tricuspid regurgitation velocity (TRV), estimated systolic pulmonary artery pressure (sPAP), right atrial diameter, right ventricular diameter, and inferior vena cava diameter are shown across increasing TAPSE/sPAP quartiles. Values are displayed on a standardized scale within each variable to facilitate comparison of trends across parameters with different units.

### 3.3 Invasive Hemodynamic Findings

The invasive hemodynamic profile closely paralleled the echocardiographic findings. Lower TAPSE/sPAP ratios were associated with substantially higher right ventricular systolic pressure, pulmonary artery systolic, diastolic, and mean pressures, and pulmonary vascular resistance (all *p* < 0.001), indicating progressively greater pulmonary vascular burden. Although cardiac output and cardiac index increased numerically across TAPSE/sPAP quartiles, these differences did not reach statistical significance. Mixed venous oxygen saturation increased significantly with higher TAPSE/sPAP ratios (*p* = 0.036), whereas pulmonary capillary wedge pressure and systemic hemodynamic parameters remained comparable among groups. Detailed invasive hemodynamic measurements are presented in Table 3, and their overall trends are illustrated in Figure 2.

**TABLE 3:** Hemodynamic parameters.

Parameter	Group 1	Group 2	Group 3	Group 4	*p* value
RAP (mmHg)	12.02 ± 8.22	9.25 ± 5.59	10.32 ± 6.13	8.95 ± 7.61	0.147
RV systolic (mmHg)	81.07 ± 26.56	59.19 ± 20.58	50.75 ± 19.50	45.32 ± 27.59	< 0.001
PAP systolic (mmHg)	80.51 ± 27.33	59.20 ± 20.22	50.07 ± 19.28	40.64 ± 20.92	< 0.001
PAP diastolic (mmHg)	28.25 ± 11.70	23.10 ± 10.52	18.86 ± 10.17	15.84 ± 9.94	< 0.001
PAP mean (mmHg)	47.76 ± 15.95	38.10 ± 13.46	31.71 ± 14.10	26.60 ± 14.24	< 0.001
PCWP (mmHg)	13.93 ± 8.27	12.73 ± 5.29	13.02 ± 6.29	12.30 ± 8.61	0.766
Aortic systolic (mmHg)	135.75 ± 30.70	142.40 ± 24.79	139.17 ± 26.74	138.03 ± 24.06	0.707
Aortic diastolic (mmHg)	72.45 ± 12.90	76.19 ± 9.53	78.29 ± 15.83	78.48 ± 11.65	0.102
MAP (mmHg)	95.70 ± 19.28	101.52 ± 13.64	101.78 ± 20.03	104.33 ± 16.21	0.148
CO (L/min)	3.91 ± 1.32	4.45 ± 1.38	4.47 ± 1.39	4.63 ± 1.08	0.112
CI (L/min/m^2^)	2.12 ± 0.56	2.39 ± 0.70	2.45 ± 0.78	2.61 ± 0.60	0.080
PVR (WU)	9.87 ± 5.70	7.43 ± 9.88	4.35 ± 3.19	3.07 ± 3.54	< 0.001
SVR (WU)	22.79 ± 6.59	21.52 ± 6.67	21.34 ± 6.70	21.78 ± 5.83	0.769
MVO_2_ (%)	53.97 ± 14.63	61.80 ± 16.00	65.41 ± 13.57	68.12 ± 19.75	0.036
FAO_2_ (%)	84.73 ± 11.06	89.55 ± 9.95	90.62 ± 8.40	92.85 ± 7.49	0.071

Abbreviations: CI, cardiac index; CO, cardiac output; FAO_2_, femoral arterial oxygen saturation; MAP, mean arterial pressure; MvO_2_, mixed venous oxygen saturation; PAP, pulmonary artery pressure; PCWP, pulmonary capillary wedge pressure; PVR, pulmonary vascular resistance; RAP, right atrial pressure; RVSP, right ventricular systolic pressure; SVR, systemic vascular resistance; WU, Wood units.

**FIGURE 2:**
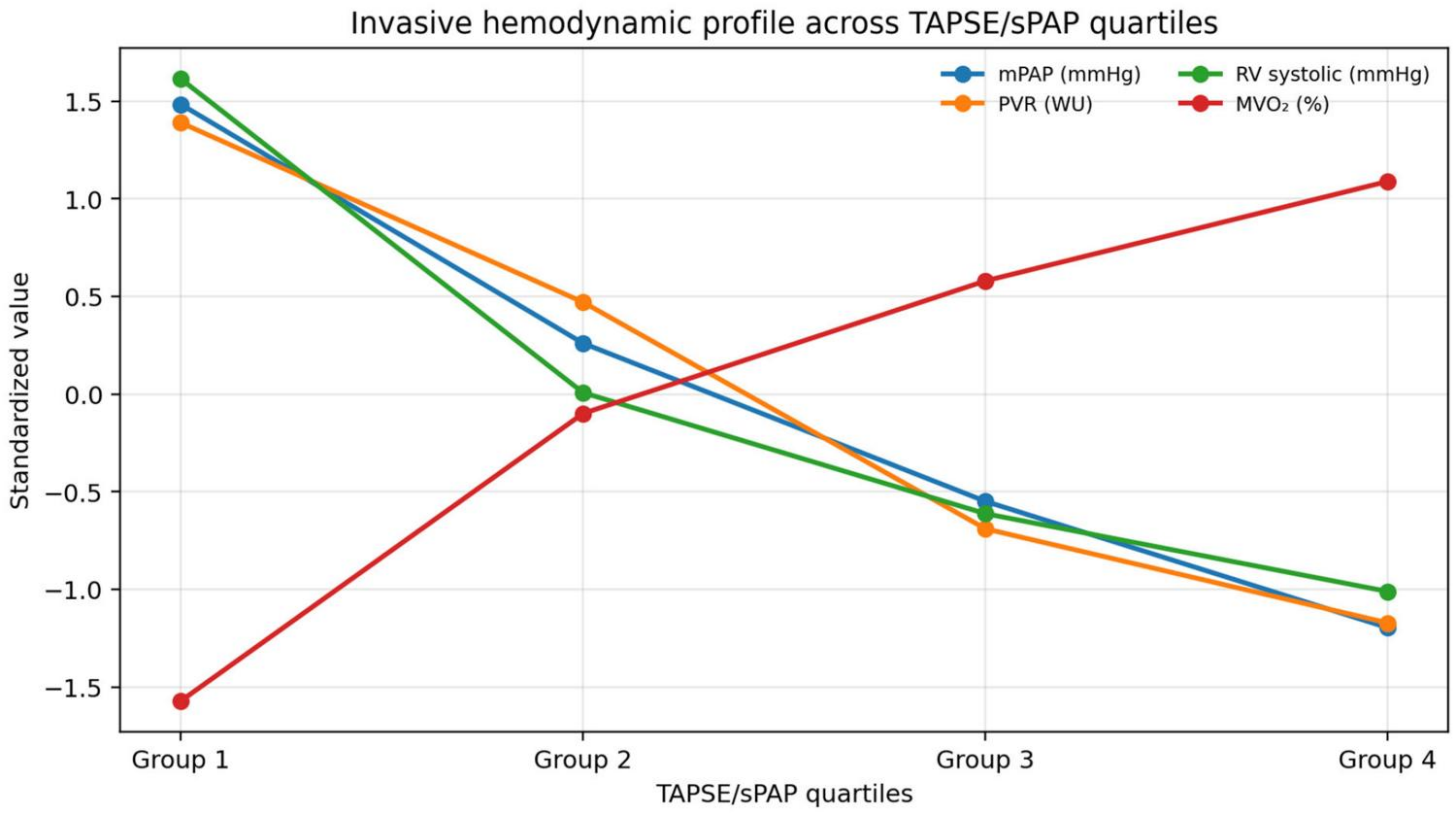
Invasive hemodynamic profile across TAPSE/sPAP quartiles. Mean pulmonary artery pressure (mPAP), pulmonary vascular resistance (PVR), right ventricular systolic pressure, and mixed venous oxygen saturation (MVO_2_) are shown across increasing TAPSE/sPAP quartiles. Values are displayed on a standardized scale within each variable to allow visualization of parallel trends despite different measurement units.

## 4 Discussion

The present study demonstrates that the TAPSE/sPAP ratio is closely associated with the clinical, echocardiographic, and invasive hemodynamic severity of pulmonary hypertension. The principal findings can be summarized as follows. First, lower TAPSE/sPAP ratios were associated with a less favorable clinical profile characterized by older age, impaired renal function, higher NT‐proBNP concentrations, and elevated serum uric acid levels. Second, progressively lower TAPSE/sPAP ratios were accompanied by more pronounced right heart remodeling and deterioration of RV systolic function on echocardiography. Finally, these structural and functional alterations were consistently paralleled by more severe invasive hemodynamic impairment, including higher pulmonary artery pressures and pulmonary vascular resistance, supporting the concept that the TAPSE/sPAP ratio represents an integrative noninvasive marker of RV adaptation to increased pulmonary afterload.

In PH, the prognostic significance of the right ventricle reflects its capacity to maintain function as pulmonary vascular load increases [9, 10]. Pulmonary artery pressure alone cannot fully characterize this ventricular response because similar pressure loads may be accompanied by substantially different degrees of RV adaptation. RV‐PA coupling provides a physiological framework for considering ventricular performance together with pulmonary vascular load. While pressure‐volume loop analysis permits direct characterization of this relationship, its invasiveness and technical complexity restrict its use in routine follow‐up. TAPSE/sPAP provides a pragmatic echocardiographic approximation of this interaction and can be derived from measurements routinely obtained during transthoracic echocardiography.

Prior studies have linked lower TAPSE/sPAP values with impaired functional capacity and adverse outcomes in PH [4, 5, 11, 12]. Our study addresses a complementary question by examining how the ratio relates to structural RV changes and invasive hemodynamic abnormalities within the same right heart catheterization‐confirmed cohort. The stepwise worsening of pulmonary artery pressures and PVR across lower TAPSE/sPAP quartiles, together with more adverse right‐heart remodeling, supports the physiological relevance of the ratio as a marker of RV‐PA uncoupling [13]. Recent evidence in systemic sclerosis further suggests that reduced TAPSE/sPAP can identify impaired RV adaptation across different stages of pulmonary vascular involvement, although reported thresholds vary by population and clinical context [7].

A key feature of the present analysis is the simultaneous characterization of clinical, laboratory, echocardiographic, and catheter‐based hemodynamic findings. This design allowed TAPSE/sPAP to be examined not simply as an outcome predictor but as an index associated with several complementary manifestations of PH severity. The observed gradients in right‐heart dimensions, RV systolic performance, pulmonary pressures, and PVR indicate that the ratio captures information spanning ventricular structure and pulmonary vascular load. This integrated pattern may help explain the prognostic associations reported for TAPSE/sPAP in previous PH cohorts [14, 15].

These findings may also have practical implications for longitudinal assessment. Right heart catheterization remains essential for establishing the diagnosis and hemodynamic phenotype of PH, but repeated invasive measurements are not appropriate or necessary at every follow‐up visit. In this setting, TAPSE/sPAP may provide complementary information regarding the degree of RV‐PA uncoupling and hemodynamic burden when interpreted alongside the broader clinical and echocardiographic assessment. It should not, however, be considered a substitute for invasive hemodynamics when catheterization is clinically indicated [15, 16, 17].

Beyond PH‐specific cohorts, TAPSE/sPAP has also shown prognostic relevance across the heart failure (HF) spectrum, in which RV dysfunction and secondary pulmonary hypertension frequently coexist. A recent systematic review including 15 observational studies and 5389 patients reported that lower TAPSE/sPAP was consistently associated with adverse outcomes across acute and chronic HF, preserved and reduced ejection fraction, valvular heart disease, infiltrative cardiomyopathies, and advanced HF [18]. Prognostically relevant thresholds were generally concentrated around 0.36‐0.40, despite substantial heterogeneity among the included populations. These observations support the concept that combining RV longitudinal systolic performance with pulmonary afterload provides information that is not captured by either TAPSE or sPAP alone. Although our study was not designed to evaluate outcomes in HF, the broader literature reinforces TAPSE/sPAP as a clinically accessible marker of RV‐PA uncoupling across cardiovascular conditions [18].

Several aspects of the present study strengthen the clinical relevance of our findings. First, all patients had pulmonary hypertension confirmed by right heart catheterization, ensuring accurate hemodynamic characterization according to contemporary diagnostic criteria. Second, we evaluated clinical characteristics, laboratory findings, comprehensive echocardiographic parameters, and invasive hemodynamic measurements within the same cohort, allowing an integrated assessment of disease severity. Finally, stratification according to TAPSE/sPAP quartiles enabled the identification of progressive changes across the clinical and hemodynamic spectrum of pulmonary hypertension, providing a comprehensive perspective on the relationship between RV function and pulmonary vascular disease.

## 5 Limitations

The present study should be interpreted in light of several limitations. First, this was a retrospective, single‐center study, which may limit the generalizability of the findings. Second, the cross‐sectional design precludes conclusions regarding causality or the prognostic impact of the TAPSE/sPAP ratio. Third, although the study included a relatively large cohort with invasive hemodynamic assessment, external validation in prospective multicenter studies is warranted. RV tissue Doppler S’ velocity and RV strain were not systematically assessed and therefore could not be included in the analysis. In addition, formal inter‐ and intraobserver variability analyses of the echocardiographic measurements were not performed. Finally, we did not evaluate longitudinal changes in the TAPSE/sPAP ratio or their relationship with clinical outcomes following PH‐specific therapies. Heart rate was not systematically available in the retrospective dataset and therefore could not be reliably included in the analysis.

Despite these limitations, the present study provides comprehensive evidence that the TAPSE/sPAP ratio reflects not only right ventricular systolic performance but also the overall pathophysiological burden of pulmonary hypertension. By integrating clinical characteristics, right ventricular remodeling, and invasive hemodynamic abnormalities into a single, readily obtainable echocardiographic parameter, the TAPSE/sPAP ratio may facilitate noninvasive assessment of RV–pulmonary arterial coupling in routine clinical practice. Future prospective studies should determine whether incorporating this parameter into multimodal risk assessment strategies improves longitudinal risk stratification and therapeutic decision‐making in patients with pulmonary hypertension.

## 6 Conclusion

The TAPSE/sPAP ratio is closely associated with clinical characteristics, right ventricular remodeling, and invasive hemodynamic severity in patients with pulmonary hypertension. Our findings demonstrate that this simple and readily available echocardiographic parameter provides a comprehensive, noninvasive assessment of right ventricular adaptation to increased pulmonary afterload. Given its strong association with both structural and invasive hemodynamic abnormalities, the TAPSE/sPAP ratio may serve as a valuable adjunct to routine echocardiographic evaluation and contribute to bedside risk stratification in patients with pulmonary hypertension. Rather than representing solely a prognostic parameter, the TAPSE/sPAP ratio emerges as an integrative noninvasive marker that reflects the clinical, structural, and invasive hemodynamic manifestations of right ventricular–pulmonary arterial uncoupling.

## Author Contributions

Contributorship: Salim Yaşar, Suat Görmel, Cemal Dinç, Ahmet Faruk Yağci, and Murat Çelik collected and analyzed data. Salim Yaşar wrote the manuscript. Özkan Eravci, Selen Eşki, Erkan Yildirim, and Murat Çelik reviewed and approved the manuscript.

## Funding

The authors have nothing to report.

## Ethics Statement

The study protocol was approved by the local ethics committee (approval no. 2026‐131) and conducted in accordance with the Declaration of Helsinki. All authors have read and approved the final version of the manuscript. Salim Yaşar, the corresponding author and manuscript guarantor, had full access to all of the data in this study and takes complete responsibility for the integrity of the data and the accuracy of the data analysis.

## Conflicts of Interest

The authors declare no conflicts of interest.

## Transparency Statement

Salim Yaşar, the manuscript guarantor, affirms that this manuscript is an honest, accurate, and transparent account of the study being reported; that no important aspects of the study have been omitted; and that any discrepancies from the study as planned (and, if relevant, registered) have been explained.

## Data Availability

The data that support the findings of this study are available from the corresponding author upon reasonable request, subject to institutional and ethics committee approval and applicable patient privacy and data‐protection requirements. The data are not publicly available because they contain information that could compromise the privacy of research participants.
